# Neurotrophin receptor Ntrk2b function in the maintenance of dopamine and serotonin neurons in zebrafish

**DOI:** 10.1038/s41598-019-39347-3

**Published:** 2019-02-14

**Authors:** Madhusmita Priyadarshini Sahu, Yago Pazos-Boubeta, Ceren Pajanoja, Stanislav Rozov, Pertti Panula, Eero Castrén

**Affiliations:** 1 Neuroscience Center, Helsinki Institute of Life Science HiLIFE, University of Helsinki, 00290 Helsinki, Finland; 2 Department of Anatomy, University of Helsinki, 00290 Helsinki, Finland

## Abstract

Neurotrophins and their receptors have highly conserved evolutionary lineage in vertebrates including zebrafish. The NTRK2 receptor has two isoforms in zebrafish, Ntrk2a and Ntrk2b. The spatio-temporal expression pattern of *bdnf* and *ntrk2b* in the zebrafish brain was studied using *in situ* hybridization. The robust and corresponding expression pattern of *ntrk2b* to *bdnf* suggests that *ntrk2b* is the key receptor for *bdnf* in the zebrafish brain, unlike its duplicate isoform *ntrk2a*. To study *ntrk2b* function, two different genetic strategies, the TILLING mutant and morpholino oligonucleotides (MO), were used. Specific subsets of the dopaminergic and serotonergic neuronal populations were affected in the mutants and morphants. The mutant showed anxiety- like behavior both in larval and adult stages. Our results consistently indicate that BDNF/NTRK2 signaling has a significant role in the development and maintenance of aminergic neuronal populations. Therefore, the *ntrk2b*-deficient zebrafish is well suited to study mechanisms relevant for psychiatric disorders attributed to a dysfunctional monoaminergic system.

## Introduction

Neurotrophins (NTs) are a family of growth factors known to play critical roles in nervous system development, maintenance, and synaptic plasticity^1,2^. The neurotrophin family includes nerve growth factor (NGF), brain-derived neurotrophic factor (BDNF), neurotrophin-3 (NT-3), neurotrophin-4/5 (NT-4/5), neurotrophin-6 (NT-6), and neurotrophin-7 (NT-7). They bind with high affinity to transmembrane tyrosine kinase proteins, Trk neurotrophin receptor kinases (NTRK),TrkA/NTRK1, TrkB/NTRK2, and TrkC/NTRK3 respectively^1^. The specific Trk receptors mediate the trophic properties of all neurotrophins. Both NTs and Trk receptors are phylogenetically highly conserved among vertebrates^3^.

The neurotrophin BDNF binds with high affinity to NTRK2 and plays a prevalent role in neuronal plasticity.Null mutants for studying BDNF or NTRK2 deficiency have been unsuccessful in rodent models due to developmental abnormalities and respiratory failure leading to postnatal lethality^4^. Most studies have been carried out on conditional knockout rodents using different cre-recombinase tagged gene specific promoters^5^. BDNF/NTRK2 signaling has been linked to both the pathophysiology of depression and the mode of action of antidepressants^6,7^. Reduction in the mRNA or protein levels of BDNF in different rodent models show attenuated antidepressant efficacy^6,8^.

The neurotransmitter serotonin has been associated with depression and is a major target for antidepressant treatment, often via the prescribed selective serotonin reuptake inhibitors (SSRIs)^9,10^. BDNF/NTRK2 molecules are co-localized in neurons of the raphe nucleus, the serotonin-producing region in the brain^11^. BDNF/NTRK2 and serotonin can co-regulate each other^9,12,13^. To understand the delay in the mode of action of antidepressants in treatment resistant patients, the bidirectional effect of these two major systems needs further investigation.

BDNF/NTRK2 also affects the reward circuitry governed by the dopaminergic circuit^2,14^. Depressive-like behavior upon BDNF alteration has been associated with the mesolimbic dopaminergic pathway^14^. The depressive-like effect produced by BDNF in the mesolimbic circuit is contradictory to its antidepressant-like effect in the hippocampus^15,16^. Further investigation on the interaction between BDNF/NTRK2 and dopamine signaling in the brain is crucial for understanding not only the effect of stress on depression, but also for addictive behavior in the functional reorganization of neuronal networks in addiction and psychiatric disorders.

An essential step for drug development largely relies on *in vivo* studies using rodents. Complete knockouts of BDNF and NTRK2 do not survive until adulthood^17,18^. Thus, this study addresses the need for a complementary vertebrate model to study the developmental aspects of BDNF/NTRK2 signaling. The zebrafish is an extensively used vertebrate model due to its high fecundity, comparable generation time as rodents and similar neuroanatomy to the mammalian brain^19^. With the advancements in knockdown and knockout techniques in zebrafish, it is increasingly used as an animal model for human biology and disease^20^. There are five Trk receptors in the zebrafish genome, trka/Ntrk1, trkb1/Ntrk2a, trkb2/Ntrk2b, trkc1/Ntrk3a, and trkc2/Ntrk3b^21^. There are two copies of Ntrk2 in zebrafish, Ntrk2a and Ntrk2b. The expression patterns of *ntrk2a* and *ntrk2b* are quite distinct from each other. The *ntrk2a* expression is more robust in the peripheral nervous system than *ntrk2b*^21^. However, the expression of each receptor isoform in the brain has required further study. Earlier studies using BDNF knockdown in zebrafish resulted in severe phenotypic abnormalities^22^. Here we demonstrate that the spatio-temporal patterning of *ntrk2b c*orresponds with zebrafish *bdnf* expression. Therefore, the BDNF receptor Ntrk2b has been the focus of this study in zebrafish. Using two alternative methods, a TILLING mutant and morpholino oligonucleotide (MO) knockdown of *ntrk2b*, we aimed to demonstrate the developmental effects of Ntrk2b function and its detrimental consequences on the aminergic systems in the larval zebrafish brain, which subsequently influence the animal’s behavior.

## Results

### Transcripts *ntrk2b*, *ntrk2a* and *bdnf* expression in zebrafish

**Figure 1:**
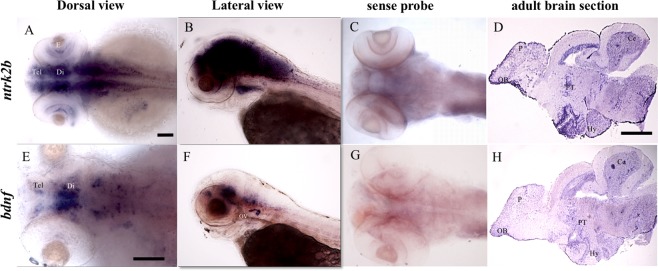
Comparative expression patterns of *ntrk2b* and *bdnf* transcripts by *in-situ* hybridization. (**A**,**B**) *ntrk2b* antisense expression at 3 dpf. Left is anterior side. (**C**) Sense probe for *ntrk2b* at 3 dpf. (**D**) *ntrk2b* expression in a 1-year-old adult brain section. Left is anterior side and top is dorsal. (**E**,**F**) *bdnf* expression in a larval brain at 3 dpf. Left is anterior side. (**G**) Sense probe for *bdnf* at 3 dpf. (**H**) *bdnf* expression in an adult brain section. Left is anterior side and top is dorsal. Tel - telencephalon, Di - diencephalon, E - eye, Hy - hypothalamus, Ce - cerebellum, PT - posterior tuberculum, P - pallium, OB - olfactory bulb. Scale bar = 100 µm.

The spatiotemporal expression of the transcripts was examined at 3 days post fertilization (dpf) and in adult brain sections by *in-situ* hybridization. The full-length *ntrk2b* mRNA was widely expressed in the brain at 3 dpf (Fig. 1A). The expression was also visible in the retina of the eye. A lateral view representation at 3 dpf shows robust expression in the brain (Fig. 1B). The sense probe served as the negative control (Fig. 1C). To characterize expression in the adult brain, sagittal sections of one-year-old adult fish brains were used for *in situ* hybridization. Expression of *ntrk2b* was observed in the dorsal telencephalon, the pallium, the parvocellular pre-optic nucleus, the posterior tuberculum, the radial glial cells lining the mesencephalic ventricle, the cerebellum, the hypothalamus, and a dispersed staining pattern in the medulla oblongata (Fig. 1D). The *bdnf* transcript at 3 dpf had a restricted expression pattern (Fig. 1E). Expression of *bdnf* is detected in the telencephalon, the pre-optic region in the diencephalon, and in the rhombomeres (Fig. 1E). Lateral view representation suggests expression in the otic vesicle at 3 dpf (Fig. 1G). The sense probe for *bdnf* served as the negative control (Fig. 1F). Similar expression pattern of *bdnf* to *ntrk2b* was detected in several regions of the adult brain (Fig. 1H). Other researchers have observed a similar expression pattern of *bdnf* in the brain^23,24^. We have also compared the expression patterns of the two isoforms of Ntrk2 by *in-situ* hybridization. The expression pattern of *ntrk2b* and *ntrk2a* was analyzed from 1 dpf until 6 dpf (Supplementary Fig. 1). The expression of *ntrk2b* was visible from 1 dpf, whereas *ntrk2a* expression was mostly undetectable at 1 dpf. At 3 dpf, *ntrk2a* expression was detected at the mid-line of the brain and in the cranial ganglia similar to previous findings by Martin *et al*.^21^. At 5 dpf, both *ntrk2b* and *ntrk2a* expression was observed in the peripheral sympathetic ganglia. Whole-mount 6 dpf brain staining of both isoforms suggests *ntrk2b* is present abundantly in the brain, unlike *ntrk2a*. Thus, *ntrk2b* expression starts early and corresponds to *bdnf* expression pattern, suggesting that it is the key receptor for BDNF in the zebrafish brain.

### Attenuated *ntrk2b* function produces no gross phenotype

**Figure 2:**
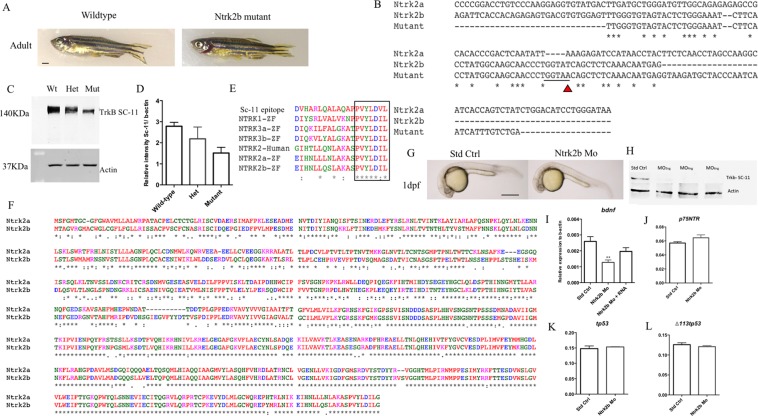
Loss of *ntrk2b* had no major effect on gross morphology, however the levels of Ntrk protein and bdnf transcript were reduced. (**A**) Gross phenotypic difference between 6-month old age-matched wild-type and Ntrk2b mutant fish. (**B**) Ntrk2b exon 17 sequence matched with the mutant sequence suggesting the point mutation causes an abrupt stop codon. (**C**) Trk protein levels from age-matched adult wild-type, heterozygous and mutant brain samples analyzed using polyclonal anti-Trk antibody. (**D**) Relative intensity values normalized to actin levels and represented as percentage of control. (**E**) Amino acid alignment of human and zebrafish Ntrk with polyclonal Trk (SC-11) antibody sequence. (**F**) Comparative protein sequence analysis of both Ntrk2a and Ntrk2b. (**G**) Gross morphology of standard control-injected and MO-injected fish at 24 hours post injection. (**H**) Trk and actin western blots of 2 dpf old MO injected samples of different doses. (**I**) The *bdnf* transcript levels in the ntrk2b morphant are significantly reduced. (**J**) The *p75ntr* transcript levels in the morphant are unchanged. (**K**) tp53 levels are unchanged in the morphant. (**L**) A tp53 isoform delta 113 transcript is unaltered in the morphant. The y-axis for the Q-RT PCR represents relative expression levels normalized to b-actin. (Bars represent Mean ± SEM, **p = 0.0029, ***p = 0,0002, one-way ANOVA.Scale bar = 100 µm).

The function of Ntrk2b in zebrafish was investigated using two different methods: a TILLING mutant and MO based translation inhibition. The Ntrk2b TILLING mutant was obtained from the zebrafish mutation project at the Wellcome Trust Sanger Institute. The mutant has a point mutation (T > A) at exon 17, which results in a premature stop codon, ultimately resulting in a non-functional Ntrk2b protein. These mutants were bred, and genotypes were grouped after sequence verification. The mutants have been outcrossed to eliminate the possible effects of non-specific mutations. The Ntrk2b adult, aged 6-month-old, mutant had no gross phenotype as compared to its wild-type littermates (Fig. 2A). The mutant sequence comparison with both the Ntrk2 isoform sequences suggests there is a stop codon in exon 17 due to the point mutation (Fig. 2B). This results in a truncated, unstable protein. Adult brains (6-month-old) were isolated from wild-type, heterozygous, and null mutant fish of both sexes and analyzed by western blotting with a polyclonal Trk antibody (Fig. 2C). Several Trk antibodies were compared, using both mouse brain lysate from the hippocampus, which served as a positive control, and a zebrafish brain sample. Only the polyclonal antibodies for Trk worked with the zebrafish sample (Supplementary Fig. 2). The specificity of this antibody in zebrafish has been verified earlier^25,26^. Compared to the wild-type protein levels, the null mutants had a significant loss of Trk protein levels normalized to actin (p-value < 0.0019) (Fig. 2D). The polyclonal antibody used for confirming the knockdown efficiency was raised against the human epitope for TRK. The epitope of the antibody sequence provided from the supplier was subjected to a multiple sequence alignment to all the five zebrafish trks and human NTRK2. The comparative sequence alignment was performed using ClustalOmega (http://www.clustal.org/omega). The C-terminal sequence appears to be conserved between human and zebrafish. The antibody detects all the five different Trk receptors in zebrafish (Fig. 2E). Thus, the remaining band observed in the mutant is likely the other Trk proteins. A comparative protein sequence alignment for Ntrk2a and Ntrk2b suggests, the kinase domain is highly conserved between the two isoforms (Fig. 2F). Furthermore, we checked the *ntrk2b* transcript levels at the exon-17 and exon 8–13 with PCR. The transcript levels were found to be unchanged. The PCR product was sequence verified to identify the mutant sequence from the wild-type and heterozygous mutants (Supplementary Fig. 3A,B).

In order to study the effects during early development, a translation-blocking MO was used. We observed no gross morphological differences between the standard control-injected and *ntrk2b* MO-injected embryos at 1 dpf (Fig. 2G). To assess the knockdown efficiency, the embryos were injected with different MO doses (2 ng, 4 ng, and 6 ng) of the *ntrk2b* MO and collected at 2 dpf. The knockdown efficiency was confirmed by western blotting using a polyclonal antibody against NTRK2/TrkB (Fig. 2H). A faint band was observed at 2 ng, while no bands were detected after the 4 ng dose. Actin served as the loading control for all the samples. We quantified the *bdnf* expression level by real-time PCR in these MO-injected animals and found a significant reduction in *bdnf* mRNA levels compared to the control-injected ones (Fig. 2I). The decreased transcription of *bdnf* could be rescued by injecting *ntrk2b* mRNA into the MO-injected animals. The p75neurotrophin receptor (p75NTR) levels were also tested and found to be unchanged in the morphants (Fig. 2J). As activation of p53 is associated with off-targeting effects caused by MO^27^, levels of tp53 and its isoform delta113tp53 were assessed to determine potential off-target effects resulting from the MOs. No change in the transcript levels of tp53 and delta 113tp53 was observed (Fig. 2K,L).

We used a MO for the other isoform Ntrk2a alone and together with the Ntrk2b MO to check if it resulted in any obvious gross phenotype. The Ntrk2a MO was a splice-blocking MO, and its efficacy was confirmed through PCR (Supplementary Fig. 3C). The morphological phenotype comparison of the injected animals with either MOs or both at 2 dpf showed no obvious phenotype amongst the groups (Supplementary Fig. 3D).

### Effect of *ntrk2b* inhibition on the dopaminergic system

**Figure 3:**
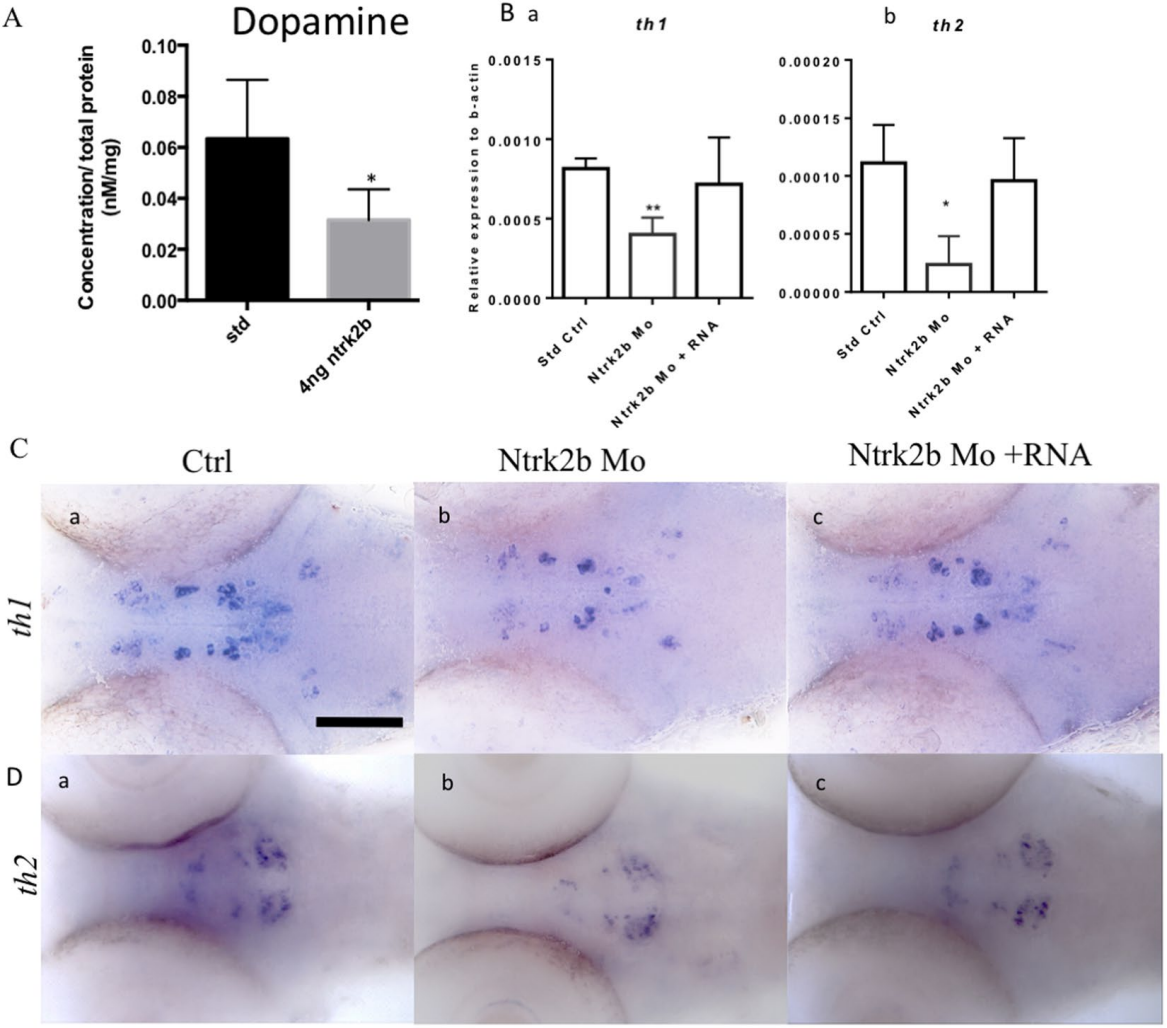
Effect of Ntrk2b deletion on dopamine and its markers at 3 dpf. (**A**) HPLC reveals total dopamine levels in the ntrk2b morphants at 3 dpf are reduced compared to controls. (**B**) (a,b) Dopaminergic neuron markers *th1* and *th2* transcripts were significantly reduced in the morphant. (**C**) (a–c) ISH of *th1* revealed loss of expression in the diencephalon. This effect could be rescued with *ntrk2b* mRNA. (**D**) (a–c) The *th2* expression was reduced in all cell populations in the morphants. The y-axis for the Q-RT PCR represents relative expression levels normalized to b-actin. (Bars represent Mean ± SEM, Student t-test *p-value < 0.05. **p < 0.001 one-way ANOVA. Scale bar = 100 µm).

BDNF/NTRK2 signaling has been associated with dopaminergic signaling in the mesolimbic circuitry in relation to chronic stress^14^. Transient inactivation of *ntrk2b* by MO reduced the total dopamine levels in the zebrafish at 3 dpf, as determined by high-performance liquid chromatography (HPLC) (Fig. 3A). There are two complementary isoforms of tyrosine hydroxylase in the zebrafish: *th1* and *th2*^28^. Quantitative estimation of the transcript levels revealed significantly reduced levels of both *th1* and *th2* in the morphants (Fig. 3Ba–b). The decreased transcript levels could be rescued by overexpression of full-length *ntrk2b* mRNA in morphants. The *th2* transcript is expressed in different cell populations from *th1*. Therefore, using whole-mount *in situ* hybridization (WISH) for the *th2 and th1* transcripts, we confirmed the PCR results and identified the dopaminergic cell populations that were affected (Fig. 3C,D). The expression of both transcripts was reduced (Fig. 3Cb,Db). This effect was rescued by over-expression on *ntrk2b* mRNA (Fig. 3Cc,Dc).

**Figure 4:**
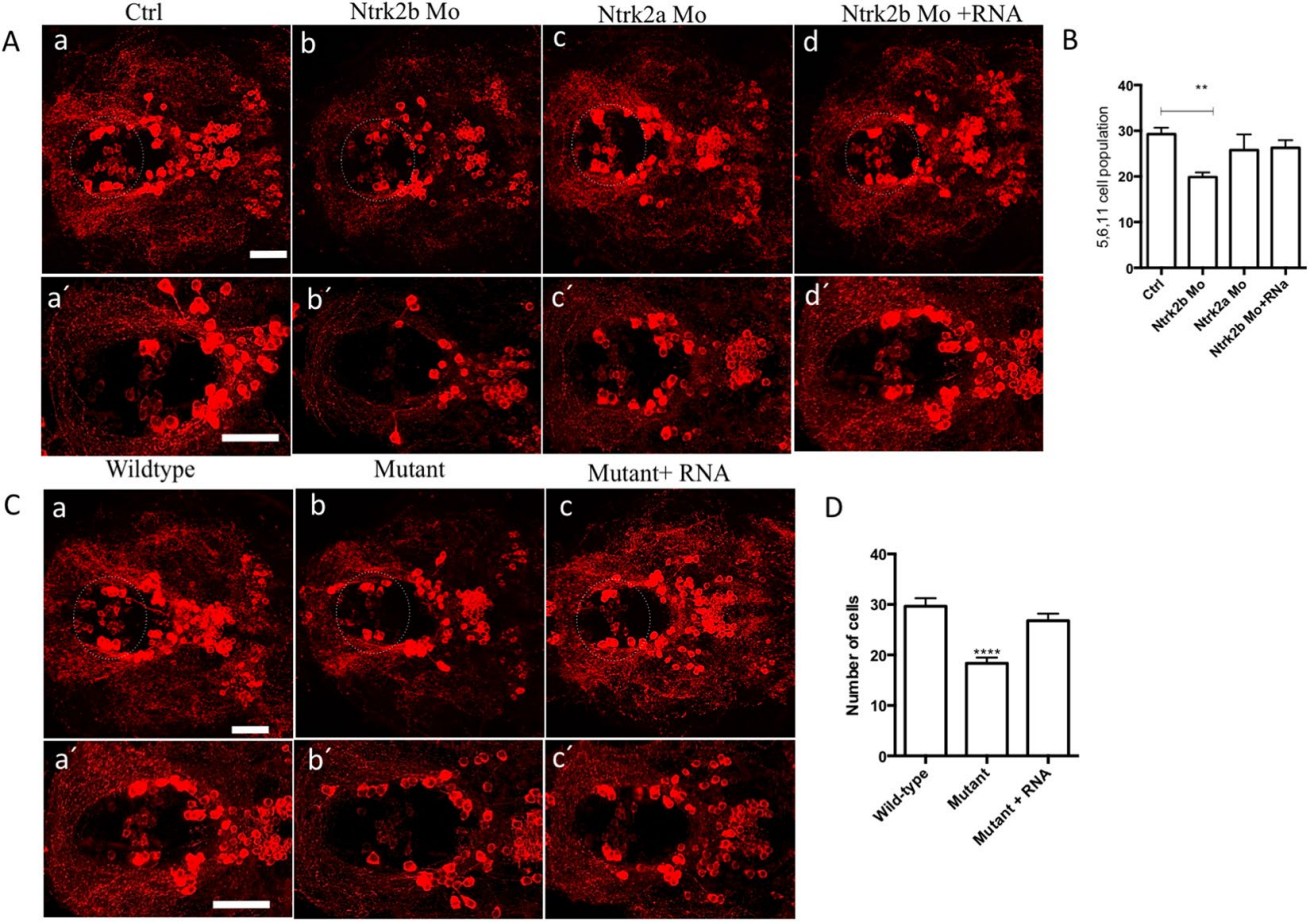
Reduced tyrosine hydroxylase immunoreactivity in both the morphants and mutants at 5 dpf. (**A**) (a–d) TH1 immunohistochemistry revealed a loss of the neuronal population in the diencephalic cluster (5,6,11 cell group) in the Ntrk2b and Ntrk2a morphant. The neuronal cell group affected has been encircled. (**A**) (a′–d′): Higher magnification images of the affected TH cell group. (**B**) Total cell counts in the diencephalic region of the morphants. (**C**) The mutants have reduced TH positive cells in the same cell group and could be rescued by mRNA overexpression. (**C**) (a′–d′) Higher magnification images of the affected TH cell group. (**D**) TH cell counts from the mutants in the 5,6,11 cell group. (Bars represent Mean ± SEM, one-way ANOVA, Kruskal-Wallis test **p-value < 0.008, ****p-value < 0.0002, Scale bar = 100 um).

To define the cell populations affected by Ntrk2b inhibition, we performed immunohistochemistry using an antibody for TH1 at 5 dpf. This antibody has been found to only recognize TH1 neurons^28^. The neuronal populations expressing the two forms of TH have been well characterized in the zebrafish^28–30^. We found that a specific ventral diencephalic population numbered 5,6,11 by Sallinen *et al*. was reduced in the Ntrk2b morphants compared to controls (Fig. 4Aa,b)^31^. No change in overall TH immunoreactive cell numbers was observed in Ntrk2a morphants (Fig. 4Ac). However, this method of numbering TH1 cell populations has been followed as it includes all groups, including those that are superimposed on each other in the horizontal representation of cell groups. The specific ventral diencephalic population numbered 5,6,11 neurons could be rescued by *ntrk2b* mRNA overexpression in the morphants (Fig. 4Ad). The images were quantified by cell counting (Fig. 4B). In the Ntrk2b mutant, the TH1 immunoreactivity was also assessed to verify the MO results at the same age. Consistently, a reduced TH1 neuronal cell population in the ventral diencephalic cluster in the homozygous mutants was observed (Fig. 4Ca–c). The cell counts from the diencephalic population revealed a significant reduction in the mutant and could be rescued by *ntrk2b* mRNA overexpression in the mutants. The pretectal, olfactory bulb, or sub pallial TH1-expressing neurons remained unchanged (Supplementary Fig. 4a). These findings were consistent in both the morphants and mutants for Ntrk2b and not for Ntrk2a.

### Effect of *ntrk2b* deletion on the serotonergic system

**Figure 5:**
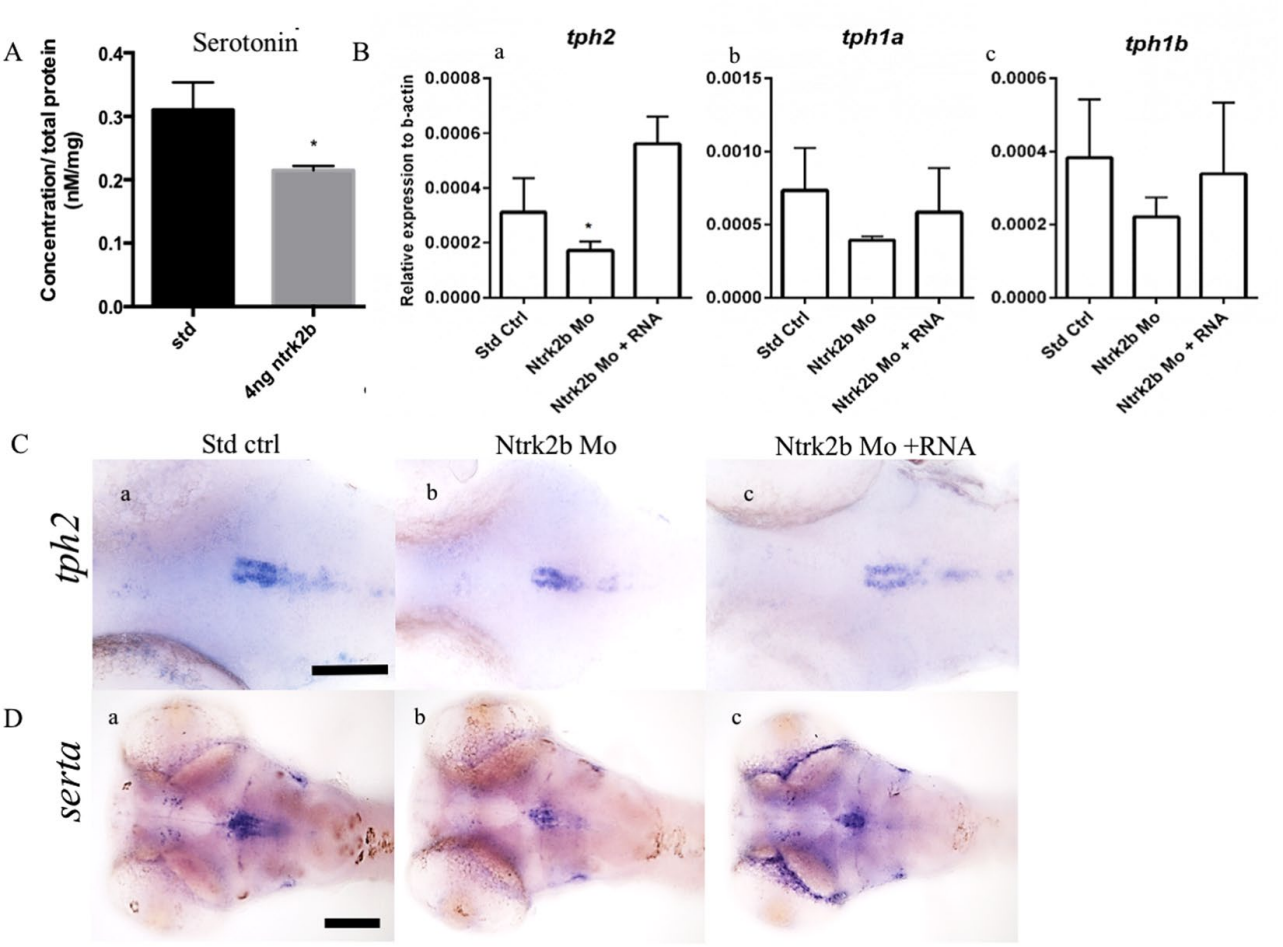
Loss of Ntrk2b effects on serotonin levels and its markers at 3 dpf. (**A**) Total serotonin levels were reduced in the morphants at 3 dpf. (**B**) The three TPH transcripts were analyzed in the morphants by QRT-PCR: Ba - tph2, Bb - tph1a, Bc - tph1b. (**B**) (a) Only tph2 was significantly reduced in the morphant and rescued by mRNA overexpression. (**B**) (b,c) The levels for tph1a and tph1b were unchanged. (**C**) (a–c) ISH with *tph2* reveals reduced levels in the morphants. (**D**) (a–c) Expression of *serta* was observed in the raphe and pretectal cluster. Levels were reduced in the morphants. The y-axis for the Q-RT PCR represents relative expression levels normalized to b-actin. (Bars represent Mean ± SEM, Student t-test *p-value < 0.05, one-way ANOVA, Kruskal-Wallis test *p = 0.025, Scale bar = 100 µm).

NTRK2 is involved in antidepressant-mediated responses, especially to selective serotonin reuptake inhibitors^32^. To elucidate the role of the loss of Ntrk2b on the total serotonin levels, we performed HPLC on the morphants. There was a significant reduction in serotonin levels in the morphants as compared to the controls at 3 dpf (Fig. 5A). The expression of the rate-limiting enzyme tryptophan hydroxylase (tph) and the serotonin transporter (Sert) is used to map the serotonergic centers in the brains of many vertebrates^33^. The *tph* levels in *ntrk2b* morphants were quantified by qPCR, and all the three isoforms were analyzed. The transcript levels of the rate-limiting enzymes *tph1a*, *tph1b*, and *tph2* were quantified (Fig. 5Ba–c). The transcript of *tph2* was significantly reduced (Fig. 5Ba). The *tph1a* and *tph1b* transcripts showed a similar trend to *tph2*. We examined the *tph2* expression in the morphants. The expression was visible in the raphe nucleus of larval zebrafish (Fig. 5C). Reduction of *tph2* expression in the morphants was rescued by *ntrk2b* mRNA overexpression. To investigate the effects of Ntrk2b on serotonin transporter levels, we used *serta*, which shares the highest homology with human and rodent *Sert*^34^. The expression of *serta* in the raphe and ventral posterior tuberculum overlaps with serotonin immunoreactivity and the expression pattern of *tph2* in zebrafish^34^. The *serta* expression at 3 dpf was visible in the raphe nuclei and ventral posterior tuberculum (Fig. 5Da–c). In the *ntrk2b* morphants, *serta* expression was invisible in the ventral posterior tuberculum (Fig. 5Db). In the raphe nuclei, *serta* expression was reduced but a few cells still expressed *serta* in the morphants. Overexpression of *ntrk2b* mRNA rescued both of the cell groups (Fig. 5Dc).

To study the serotonergic cell groups that were affected, the larval brains at 5 dpf were immunostained using a serotonin-specific antibody that has been well characterized in the zebrafish^35,36^. In comparison with mammals, there are five complexes of serotonin cell groups in the zebrafish: the pre-tectal/thalamic complex, the hypothalamic periventricular complex, the rostral raphe complex, the caudal raphe complex, and the area postrema complex.

**Figure 6:**
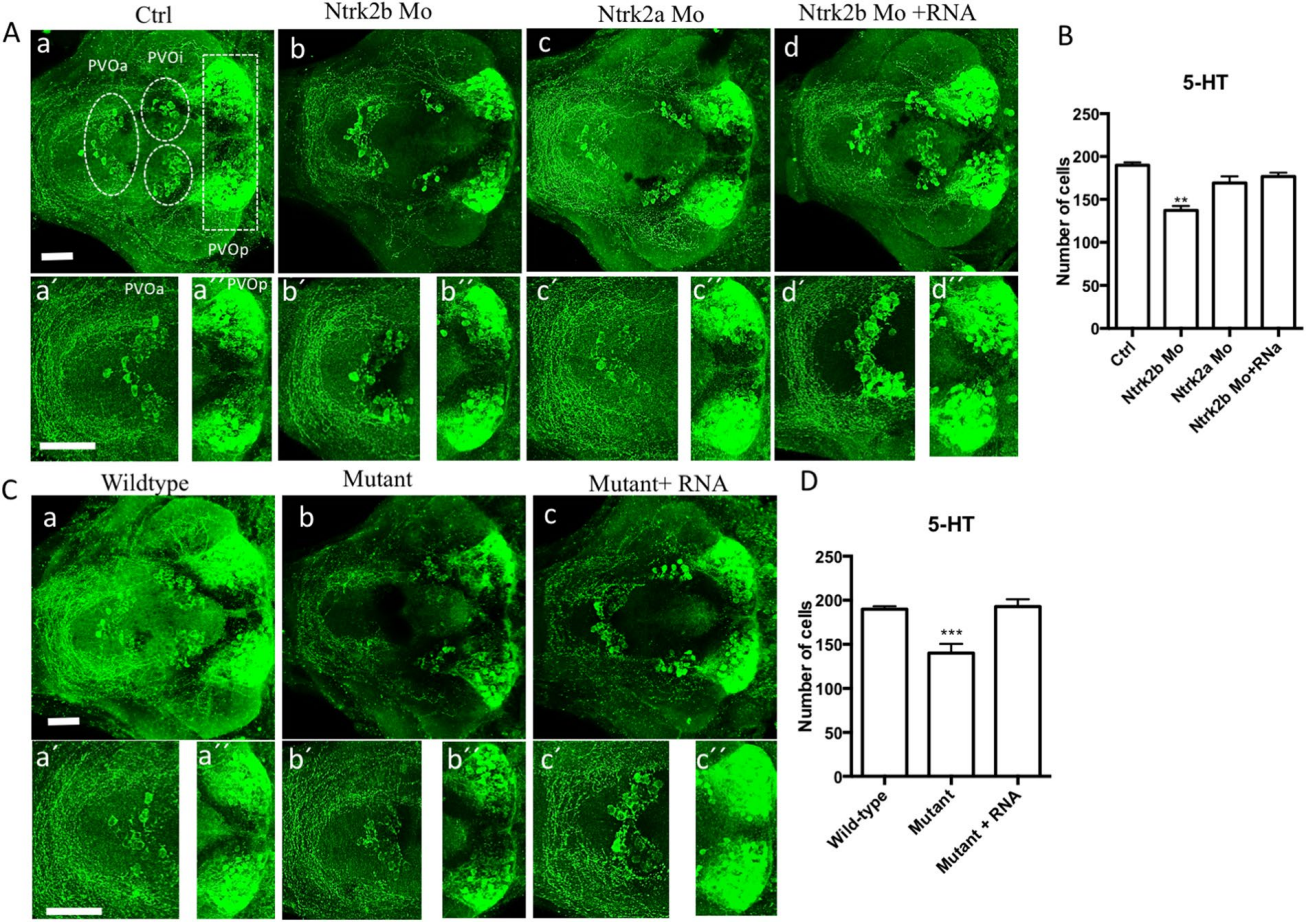
Immunoreactivity of serotonin was altered in the morphants and mutants at 5 dpf. (**A**) (a–d) Serotonin immunoreactivity was reduced in the morphant. (a) Ctrl. (b) Ntrk2b MO. (**C**) Ntrk2a MO and (d) Ntrk2b + mRNA. (**B**) Total serotonin positive cell counts shows significant reduction in the Ntrk2b MO. (**A**) (a′–d′) Higher magnification images of the PVOa and (**A**) (a′′–d′′) Higher magnification images of the PVOp. (**C**) The Ntrk2b mutants had reduced serotonin immunoreactivity as compared to wild-type littermates (a) wild-type, (b) Mutant, (c) Mutant injected with *ntrk2b* mRNA. The expression of 5-HT in the mutant is aberrant. (**C**) (a′–d′) Higher magnification images of the PVOa and (**C**) (a′′–d′′) Higher magnification images of the PVOp. (**D**) Comparative cell counts from the three groups representing reduced cell numbers in the mutant. PVOa – paraventricular organ, anterior part, PVOi – paraventricular organ, intermediate part, PVOp – paraventricular organ. (Bars represent Mean ± SEM, one-way ANOVA. **p-value < 0.002, ***p-value < 0.0004). Scale bar = 100 µm.

We observed a significant reduction in 5-HT immunoreactivity in the morphants compared to the controls (Fig. 6Aa–d). There was a concomitant loss of neuronal cells and axonal projections in the morphants was detected (Fig. 6Ab). This loss could be rescued by overexpression of *ntrk2b* mRNA (Fig. 6Ad). Interestingly, no change in serotonin immunoreactivity was observed in ntrk2a morphants (Fig. 6Ac). For cell counting, we focused on the serotonin populations in the diencephalic region: the periventricular organ anterior part (PVOa), periventricular organ intermediate part (PVOi) close to the midline in the posterior tuberculum, and the periventricular organ posterior (PVOp) in the ventrocaudal hypothalamus. Total cell counts of serotonin immunoreactive cells in these morphants were analyzed. The *ntrk2b* morphants had significantly reduced cell numbers (Fig. 6B).

In the *ntrk2b* mutant, 5HT immunoreactivity in the larval brain at 5 dpf was assessed. We observed a reduction in all 5HT cell groups in the larval mutant brain (Fig. 6Ca–c). The affected cell groups could be recovered by mRNA injection. The effects of Ntrk2b loss were observed in different serotonergic cell populations analyzed by cell counting (Fig. 6D). Whole-mount 5 dpf brain serotonin immunoreactivity in all the different groups is represented in Supplementary Fig. 4B. Thus, the reduced serotonin immunoreactivity observed in the morphants and mutants for Ntrk2b was similar, but no significant change was observed in Ntrk2a morphants.

### Anxiety like behavior in Ntrk2b mutants

A substantial number of transgenic mice or rats with modified BDNF or NTRK2 signaling have been produced to mimic anxiety and depressive behaviors^8^. The conditional mutants produced with cre-flox recombination in rodents for BDNF and NTRK2 have also been associated with anxiety and depressive behaviors^16,37^. Inconsistencies in the reported behavior of these transgenic rodent models have greatly hampered any mechanistic studies linking the cause or effect of these important molecules in mood disorders and depression. Currently the zebrafish are being robustly used for developmental, genetic, and drug testing studies. Several behavioral paradigms using zebrafish have been designed and have successfully tested anxiety-like behavior^38,39^.

**Figure 7:**
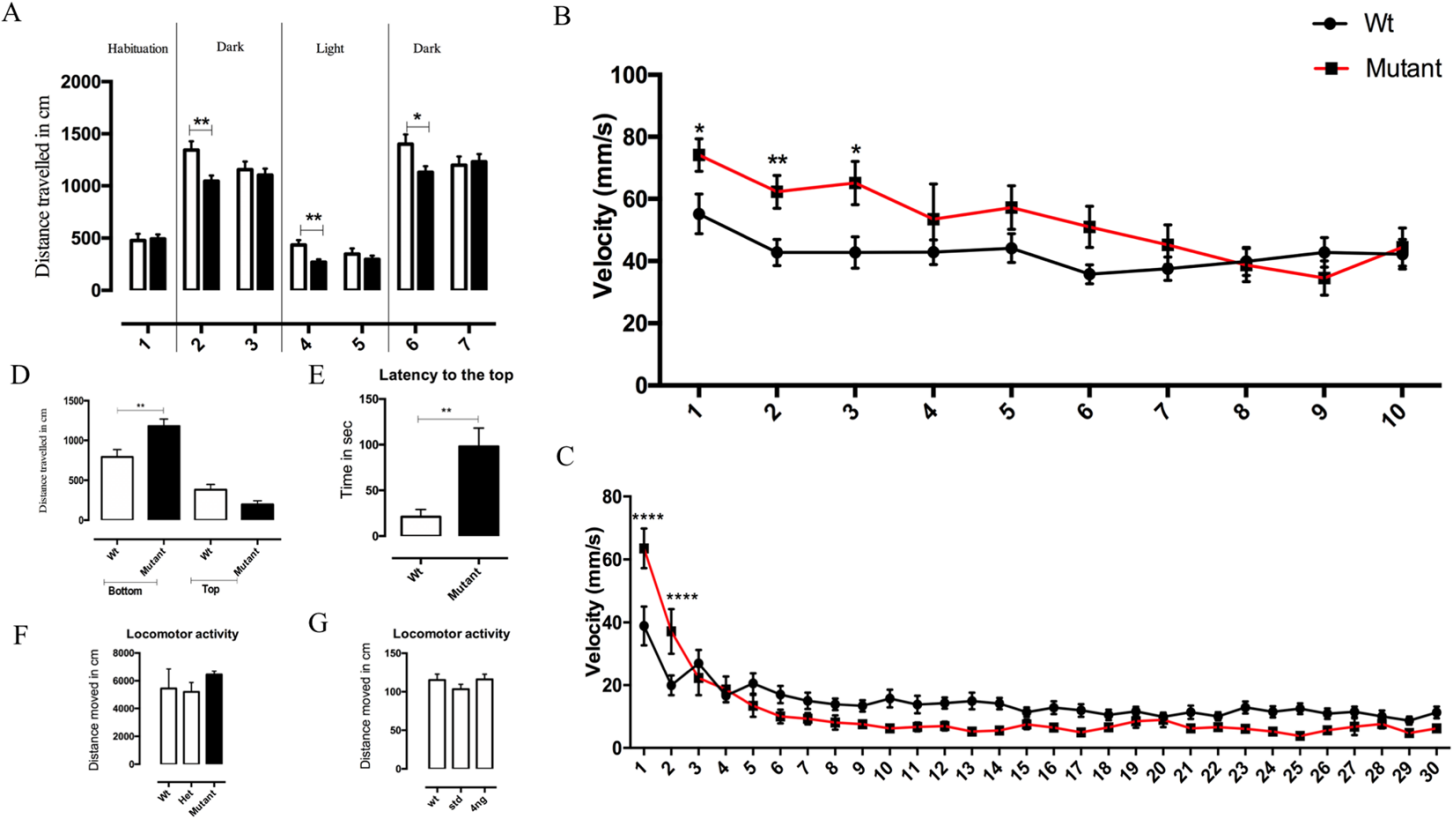
Anxiety-like behavior in Ntrk2b mutants. (**A**) Dark- light -dark activity test of 6 dpf Ntrk2b mutant larvae with 10 mins interval between the sessions. Each session was divided into blocks of two. (**B**) Acoustic startle responses of 6 dpf Wt and mutant larvae. (**B**) (a) Mean value of the maximum velocity during 10 stimuli with a 20 s ISI. (**C**) Analysis of acoustic startle response during 30 stimuli with a 1 s ISI. (**D**) Novel tank diving test using Ntrk2b adult mutants 9 months old. Distance travelled in the bottom of the tank by the mutant is significantly higher. (**E**) Latency to move to the top of the tank is higher in the mutants. (**F**) Locomotor activity of Ntrk2b adult mutants. (**G**) The locomotor activity of Ntrk2b morphants larvae. (Bars represent Mean ± SEM, two-way ANOVA with repeated measures: *p < 0.05, **p < 0.01, ***p < 0.001; ISI- inter stimulus interval).

Therefore, we tested the behavior of the Ntrk2b mutants to study anxiety. We first tested the the larval mutant animals 6 dpf old using the dark-light-dark behavioral assay. The total distance moved parameter was measured across different time blocks. The zebrafish larvae moved significantly more during the dark period than during the bright light period, in line with previously published results^38^. During habituation, the mutant’s movement was comparable to the control fish (Fig. 7A). When the dark phase was divided into two blocks of 5 mins each, the mutants were significantly less active during the first 5 mins after the lights went off (p < 0.01). The activity normalized in the subsequent block of dark phase. In the light period, the activity of the mutants was again reduced for the first block of 5 mins (p < 0.01) and normalized later. Similarly, in the last session from the light to dark transition, the mutants again moved less during the first 5 mins (p < 0.05). Therefore, the activity of the mutants was reduced every time the dark-light transition occurred and then normalized in the subsequent block.

To study startle response in zebrafish, the tap test has been used as described previously^38^. In this acoustic/vibrational behavior test, the larvae responded to the stimuli, a tap, at an intensity above the baseline level. The mean startle response to 10 stimuli at 20 s interval for 200 s termed as inter stimulus interval (ISI), was represented as the maximum velocity (mm/s) parameter for both groups. The results suggested that larval Ntrk2b mutants 6 dpf show an initial increase in the startle responses and then habituate to repeated stimulus presentation similar to the level of wild-types (Fig. 7B). In the startle response for 30 stimuli at a 1 s ISI, the mutant fish showed a similar difference in their behavior compared to controls: initially an exaggerated response to the first two stimuli followed by a decrease to the wild type fish levels (Fig. 7C). This was analyzed using a two-way repeated measures ANOVA.

The behavioral responses in the larvae are not exactly comparable to the adults^40^. Therefore, we tested whether the mutants’ anxiety-like behaviors persisted into adulthood. We used a novel tank diving test with adult zebrafish mutants and age-matched wild-type fish 9 months old (Fig. 7D). Wildtype and mutant fish of both sexes were used in this test as no significant differences between the genders was observed. The mutants showed a stronger preference for the bottom compartment compared to the top of the tank (Fig. 7D). The total distance travelled by the mutants in the bottom of the tank was significantly longer than that of the wild-types. The latency to exit to the top of the tank was higher in the mutants (Fig. 7E). This suggests increased anxiety-like behavior of the mutants in a novel environment. The overall motor activity of the mutants was normal (Fig. 7F). Similarly, the overall motor activity of morphants and the controls was also found to be normal (Fig. 7G).

## Discussion

In the current study, we established that the major receptor for BDNF in the zebrafish brain is Ntrk2b. Due to the limitations of MO methods^41,42^, we used both morphants and Ntrk2b null mutants to analyze *ntrk2b* function in the maintenance of the two major aminergic systems. The MO knockdown method was verified using appropriate controls and serves as an additional method which does not suffer from poorly understood genetic compensation which can affect the TILLING mutant experiments. Furthermore, we show that the loss of Ntrk2b had effects on the fish behavior during development as well as in adulthood.

For this study, we considered both the isoforms and their effects on monoamines, especially dopamine and serotonin. Ntrk2a morphants had no effects on the gross phenotype or on the aminergic systems, while Ntrk2b morphants and mutants showed substantial defects. The two duplicated isoforms of Ntrk2: *ntrk2a* and *ntrk2b*, expression patterns have been studied earlier^21^. A comparison of their expression pattern to that of BDNF has not been reported. The expression of *ntrk2b* was detectable at 1 dpf, while detection of the *ntrk2a* expression was not observed until 3 dpf. The expression of both *bdnf* and *ntrk2b* were comparable to the patterns observed by several different groups^23,43^. Thus, Ntrk2b is the major receptor of BDNF in the zebrafish brain and its function is essential in maintaining two important aminergic systems involved in anxiety, stress and depression: the dopaminergic and the serotonergic systems.

The sequence homology for *ntrk2b* in zebrafish with that of humans and mice is 62%. The zebrafish retains about 20% of duplicated gene pairs. The absence of complete knockout mutants for both BDNF and NTRK2 in rodents has been a limitation in understanding the effect of the BDNF/NTRK2 signaling pathways during development. Interestingly, the viability of the *ntrk2b* zebrafish null mutant could aid in understanding cell type specific roles of this receptor. The increased viability of the *ntrk2b* mutant zebrafish is unlikely due to compensatory effects of its duplicated gene *ntrk2a*, because the double knockdown of both the isoforms for NTRK2 did not affect the survival or the phenotype of the zebrafish over that observed in Ntrk2b mutants. With the currently available antibodies that may show some degree of cross-reactivity with other NTRKs, we cannot completely rule out the possibility that small amounts of a full-length or truncated NTRK2b expression might remain in our morphants and mutants. Therefore, it is possible that our fish represent hypomorphs rather than complete knockouts, which might contribute to the improved viability. However, we observed that the loss of Ntrk2b resulted in an aminergic phenotype in zebrafish, which has not been detected in the mammals, which indicates a differential role for NTRK2 in fish and mammals. Although the evolutionary distance between zebrafish and mammals is considerable, comparing the brain development could reveal crucial regulatory elements. This novel finding could be beneficial in deciphering the physiological processes underlying effective antidepressant mechanisms or psychiatric disorders involving BDNF/NTRK2 signaling.

In the Ntrk2b-deficient zebrafish, the dopaminergic cell population in the ventral diencephalic cluster, which corresponds to the mid-brain dopaminergic cell groups A9/A10 in mammals^30^, was reduced in number in both mutants and morphants. This effect could be reversed by overexpression of the full-length *ntrk2b* mRNA. In contrast, deletion of the duplicated gene ntrk2a did not affect the TH cell populations. Brain-derived neurotrophic factor (BDNF) is known to exert a trophic effect on the mesencephalic dopaminergic neurons^44^, and *in vitro* studies on mesencephalic dopamine neurons suggest that the effect of BDNF via NTRK2 receptor activation is responsible for dopamine release^45^. These neuronal populations have been linked with several neurological disorders such as Parkinson’s disease, schizophrenia, addiction, and psychomotor retardation.

BDNF and its high-affinity cognate receptor, tyrosine kinase B (NTRK2), have been linked to neurochemical and behavioral responses of serotonin (5-HT) following SSRI treatment. Therefore, we investigated the serotonergic neuronal cell groups in these mutants. Serotonin cell populations have been very robustly studied and characterized in the zebrafish. Unlike the restricted effects of Ntrk2b deletion on a subset of dopaminergic neurons, all the serotonin populations were reduced in both mutant and morphant fish. In zebrafish there are three *tph* genes, *tph1a*, *tph1b*, and *tph2*. Only the levels of *tph2* were significantly reduced, while the two other isoforms showed a trend towards reduction. In the Ntrk2b deficient fish, serotonin synthesis as well as total serotonin were reduced. Previous studies have clearly linked an autocrine/paracrine feedback loop operating the effects of serotonin on BDNF/NTRK2 signaling, and BDNF/NTRK2 signaling is crucial for the development of serotonergic neurons^13^. In addition, the markers for serotonin synthesis and re-uptake, *tph2* and *serta*, were significantly reduced in the raphe, suggesting a direct role of *ntrk2b* in serotonergic neuron development, reuptake and maintenance. These results suggest an important regulatory mechanism between the BDNF/NTRK2 and serotonin. Thus, these mutants could potentially be used to dissect the complex neuronal circuitry associated with mood-related behaviors.

Dopaminergic and serotonergic neuronal systems have been linked to anxiety and depression^9^. While the general locomotor behavior in both the morphants as well as the mutants remained unchanged, in the behavior paradigms for anxiety and stress, the mutants showed an anxiety like behavior both during development and in adulthood. The mutant larvae showed an initial exaggerated response to novel environmental conditions, but upon repeated stimulation, they showed a freezing-like behavior with reduced movement when compared to control fish. This freezing-like behavior observed in the altering light conditions and acoustic/vibrational test in our mutants is consistent with altered serotonin signaling. Interestingly, the adult mutants also showed higher anxiety levels in a novel environment. The increased latency to exit to the top of the tank and time spent exploring the bottom of the tank has been shown to be related to an anxiety-like phenotype in adult zebrafish. Thus, the Ntrk2b mutant produces an anxiety-like profile both during development and in adulthood. Therefore, zebrafish could serve as a good model for understanding the biochemical and physiological role of Ntrk2.

## Materials and Methods

### Zebrafish strain and maintenance

The zebrafish strain used in the experiments was the wild-type Turku strain. It has been maintained in our facility for more than a decade^28,31,36^. Animals were raised at 28 °C and staged as described earlier^40^. The mutant for Ntrk2b (Sa13660) was obtained from the zebrafish mutation project (Wellcome Trust Sanger Institute) and outcrossed with the wild-type Turku strain at least two times. For all experiments fish of both the sexes were used. It is not possible to detect the gender of fish during embryonic days such as 1–6 days post fertilization (dpf); therefore, the pooled cohort of the fish was used for all the subsequent experiments. In each experiment, the number of larval animals was n = 30/group. Each experiment was repeated at least three times unless stated otherwise. Whenever adult zebrafish were used, the gender was identified and compared before pooling them into groups. Adult zebrafish used in the experiments were aged between 6–12 months. The exact age is mentioned in each experiment. All experimental procedures were performed in accordance with institutional animal welfare guidelines and were approved by the Office of the Regional Government of Southern Finland in agreement with the ethical guidelines of the European convention (ESAVI/10300/04.10.07/2016).

### Cloning *ntrk2b and ntrk2a* constructs

The full-length *ntrk2b* was synthesized using the following primers:

F: GGATCCCGCTAGACCTGCTATGACCG, R: GGATGTCCAGGTACACAGGCCCTAGG. A fragment of *ntrk2a* was synthesized using the following primers: F: GCCTCAGAAACCTAACCGTCA, R: GAGGTCCAAGTGGAGTGTCG. The PCR cycling parameters were 94 °C for 2 min and 40 cycles of 94 °C for 30 sec, 58 °C for 30 sec, 72 °C for 2 min, followed by an extension at 72 °C for 10 min. The PCR fragment was cloned into PGEMT-easy vector systems according to the manufacturer’s instructions (Promega, Madison, WI) and was verified by sequencing. The bdnf clone contains a partial fragment of the zebrafish transcript. The primers used for cloning the bdnf fragment were F: CCCTCGCTCACGGACACTTT R: CGAGTTATAGTGCCGCTTGTCT. The sequence of bdnf is attached in the supplementary file (Supplementary Fig. 5).

### MO design and mRNA rescue injections

The antisense MOs were ordered from GeneTools LLC. The 5′UTR was targeted for designing the Ntrk2b MO (5′-TTCCACGAACCCCTGCGGTCATAGC). We have also tested another splice MO (GCAACCTAATGCAAACCCAAACAGA) targeted at intron 4 and exon 5 (i4e5MO) for Ntrk2b. The Ntrk2a MO was designed at a splice site in the exon-intron boundary i5e5 (5′-GCAGCCTGCAAATATACGACCACAT). The MO solution comprised of 25% phenol red and MO in sterile water and was injected in a 4 nl volume to each embryo. The working concentration of the MOs was determined by injecting different dose of MO. The Ntrk2b MO dose was 4 ng, i4e5MO was used at 12 ng and Ntrk2a MO was 8 ng. A standard control MO was used as an injection control^46^. A control morpholino oligonucleotide (MO) having a sequence (5′-CCT CTT ACC TCA GTT ACA ATT TAT A 3′) was used as a standard control. All experiments for Ntrk2b were done using the 5′UTR targeting translation blocking MO.

The full-length *ntrk2b* sequence was custom-made, sequence matched and codon optimized without any mutations (Geneart^TM^ Invitrogen, Thermo Scientific Fisher, Carlsbad, USA). Additional restriction sites were inserted to subclone it to the pMC vector containing untranslated repeats and a polyadenylation site, which can enhance mRNA stability and translation efficiency. The pMC expression vector was kindly provided by Dr Thomas Czerny^47^. The pMC vector containing the full-length *ntrk2b* insert was linearized with NotI, and capped full-length transcripts were generated with mMESSAGE mMACHINE kit (Ambion, Austin, Tx) using T7 polymerase. The synthesized mRNA was measured by a Nanodrop 2000c (Thermo Fisher Scientific, USA) before injection. For the mRNA rescue experiments, 1 ng of full-length *ntrk2b* mRNA was co-injected with 4 ng Ntrk2b MO at the one-cell stage embryos. The mutants were co-injected with 1 ng of full-length mRNA for rescue experiments.

### *In-situ* hybridization

Fish at different stages from 1–6 dpf were grown in the embryo medium with PTU (1-phenyl 2-thiourea). They were fixed in 4% PFA in phosphate buffered saline (PBS) o/n at +4 °C. The fixative was changed the next day to 100% methanol and the samples were stored at −20 °C until further use. The 6 dpf brains were dissected and stored in similar manner with 100% methanol. The adult brains 1 year old (n = 6) were dissected and fixed with 4% PFA o/n at +4 °C. They were embedded in cryo-embedding mix until solidified. The brains were sectioned at 14 μm on Superfrost slides and kept at −80 °C until further use. The *in situ* hybridization method applied has been described earlier^48^. The probes were synthesized from the clones *serta* (a kind gift from K. Shirabe)^34^, *th1*, *th2*, *tph2*, *ntrk2b*, *ntrk2a* and *bdnf*. The probes for *th1* and *th2* has been described in our previous publications^28^. The probes for *ntrk2b*, *ntrk2a* and *bdnf* were synthesized from the clones mentioned earlier. The larval fish n = 12/group was used with each probe. The digoxigenin (DIG)-labeled probes were generated with the DIG RNA labeling kit according to the manufacturer’s instructions (Roche, Mannheim, Germany). The samples were mounted in 80% glycerol and brightfield images were taken using a Leica DM IRB inverted microscope with a DFC 480 charge-coupled device camera. The z-stacks were processed with Leica Application Suite software (Leica microsystems, Mannheim, Germany).

### Western blotting

The adult mutant zebrafish brains (n = 4/group) of 6 months old and age-matched wild-type adults were dissected. The MO-injected fish and control injected fish (n = 30/group) at 2 dpf were pooled and collected for sampling. Samples for protein extraction were processed in RIPA lysis buffer (3 M Tris HCl pH 8, 5 M NaCl, 0.5 M NaF, NP-40, glycerol, and protease inhibitor cocktail tablets). Homogenized fish were centrifuged at 13 000 rpm for 15 minutes at +4 °C. The supernatant was collected and protein measurement was performed using the DC assay kit (Biorad, USA). An equal amount of protein was loaded onto an SDS-PAGE gel and blotted on a PVDF membrane following incubation with the antibodies. The membranes were stripped and re-probed with housekeeping genes for normalization. The list of  primary antibodies used are described in Supplementary Table 2. The secondary antibodies were hrp-conjugated goat anti rabbit for TrkB SC-11 and GAPDH and goat anti mouse for actin. The membrane was developed by chemiluminescence using the Pierce ECL kit (Thermo Fisher scientific, USA) followed by imaging using a Fuji LAS-3000 Camera (Tamro Medlabs, Vantaa, Finland).

### Quantitative real-time PCR

Total RNA was isolated from pooled whole zebrafish larvae 3 dpf using a PureLink® RNA Mini Kit (Thermo Fisher scientific, USA). In each experiment, there were 30 animals per group in triplicates. The RNA was reverse transcribed using the SuperScript IV reverse transcriptase (Invitrogen/Thermo Fisher scientific, USA) primed with random primers according to the manufacturer’s instructions. The real time PCR was carried out in the CFX96 Touch Real-Time PCR detection system (Biorad, USA) instrumentation with SYBR master mix (Thermo Fisher scientific, USA). The reaction mixture comprised of: 12.5 ul of SYBR green master mix, 1–3ul of cDNA and primers at a final concentration of 1 uM. The data were calculated by the comparative method using Ct values of β-actin as the reference control. The list of primers used were compiled in the supplementary file (Supplementary Table 1). The results were analyzed using GraphPad Prism software (GraphPad Software, Inc. CA, USA). The *ntrk2b* mutant transcripts was analyzed using primers at exon 17 and at exon 8–13.

### Immunostaining and cell counting

All the fish embryos were fixed at 5 dpf with 4% PFA overnight at +4 °C. The larval brains were dissected with a stereo microscope, n = 7/group in triplicates. The samples were processed as published earlier^28,36^. The primary antibodies used were anti-tyrosine hydroxylase monoclonal mouse antibody and anti-serotonin rabbit antibody at 1:1000 dilution. The secondary antibodies were Alexa Fluor® 488 or 568 goat anti-mouse or anti-rabbit IgG(Thermo Fisher scientific, USA) . Immunofluorescence samples were mounted in 80% glycerol and examined under a Leica TCS SP2 AOBS confocal microscope (Leica microsystems, Mannheim, Germany). For excitation, an argon laser (488 nm) and a diode laser (568 nm) were used. The emission was detected at 500–550 nm and 550–650 nm respectively as described earlier^36^. Stacks of images taken at 1.2-μm intervals were compiled, and final images were produced with Leica Confocal Software using the maximum intensity projection algorithm. The stacked images were imported to the open access Fiji software for cell counting^49^. Statistical analysis of the cell counting was performed using One-way ANOVA.

### Amine levels measurement

For the analysis of dopamine and serotonin levels, 30 whole larvae were sonicated in 150 μl of 2% perchloric acid and centrifuged for 30 min at 15,000 g, after which 10 μL of the filtered supernatant was injected into a high-performance liquid chromatography (HPLC) system equipped with a Waters Concorde electrochemical detector (Waters, Milford, MA, USA) set to a potential of +0.80 V, a column oven and a column Gemini C18 5 μm 150 × 4.60 mm (Phenomenex, Torrance, CA, USA). The mobile phase consisted of purified water with 8% methanol, 50 mM citric acid, 1.5 mM 1-octanesulfonic acid, 0.05 mM EDTA, and 50 mM phosphoric acid. The column temperature was set at 37 °C and the flow rate at 1 ml/min. System control, data acquisition and analysis were performed using Waters Empower software (Waters, Milford, MA). Concentrations of the catecholamines and metabolites were calculated from standard curves which were linear from 10 nM to 1 μM. To normalize the data, sample protein concentration was measured using the Pierce© BCA Protein Assay Kit (Thermo Fisher Scientific INC., Rockford, IL, USA). Three individual groups per treatment condition were measured by a blinded experimenter.

### Behavioral analysis

For behavior analysis, larval zebrafish at 6 dpf were used. Identification of different sexes is not possible at the stage used for analysis. The pooled fish of mutants and age-matched wild-type groups were habituated individually, one fish per well, in a 48-well plate for at least 10 mins before tracking. For tracking, 24 fish of each strain were used. The activity was monitored and analyzed using Danio Vision system (Noldus, Netherlands). The data were analyzed using GraphPad Prism software (GraphPad Software, Inc.CA, USA). The protocols for light-dark and acoustic startle in larvae were derived from van den Bos *et al*.^38^. We analyzed the total distance moved (mm) during the 5 min time interval for general activity under light and dark conditions. For acoustic/vibrational startle we analyzed the maximum velocity (mm/s) variable during the inter-startle stimulus (ISI). This parameter captured the short burst of activity or startle response better. The startle response was studied at 20 s ISI and 1 s ISI.

In the novel tank diving test, 9 months old adult mutant zebrafish and age-matched wild-type controls were used. The animals were separated based on the sex and genotype into 4 different tanks. No baseline change was observed between the different sexes. Therefore, we pooled the animals into two groups during analysis. A total of n = 12 per group was used for this study. The animals were habituated for 30 min in the room. Each animal was tracked for a total of 6 min. The tracking was analyzed using Ethovision XT version 13 (Noldus, Netherlands). Several parameters such as total distance moved, latency to reach the top of the tank and transition frequency in between the top and bottom of the tank was measured.

### Statistical analysis

All the experiments were analysed using GraphPad Prism software (GraphPad Software Inc. CA, USA). Student’s t-test was used when two groups were compared or when appropriate. For more than two groups, the analyses were performed using one-way analysis of variance (ANOVA) followed by Tukey’s post-hoc test. For the light-dark and acoustic stimulus, data analyses were done with a two-way repeated measures ANOVA followed by Sidak’s multiple comparisons test. All the error bars represent mean ± SEM. The significance value was accepted at p ≤ 0.05.

## Supplementary information

Supplementary information
