# Supplementary material for: Neurotrophin receptor Ntrk2b function in the maintenance of dopamine and serotonin neurons in zebrafish
Source: Sci Rep. 2019 Feb 14;9:2036. doi: 10.1038/s41598-019-39347-3 (PMC6375947; doi:10.1038/s41598-019-39347-3)
Supplement: Supplementary file 1 — Supplementary information [file 41598_2019_39347_MOESM1_ESM.docx]

**Neurotrophin receptor Ntrk2b function in the maintenance of dopamine and serotonin neurons in zebrafish.**

Madhusmita Priyadarshini Sahu^1^, Yago Pazos^1^, Ceren Pajanoja^1^, Stanislav Rozov ^1^, Pertti Panula ^1,2^ and Eero Castrén ^1*^.

1 Neuroscience Center, Helsinki Institute of Life Science HiLIFE, University of Helsinki, 00290 Helsinki, Finland

2 Department of Anatomy, University of Helsinki, 00290 Helsinki, Finland

Supplementary table 1- List of primers for Q-RT-PCR

| Zf b-actin 1F | CGAGCAGGAGATGGGAACC |
| --- | --- |
| Zf b-actin 1R | CAACGGAAACGCTCATTGC |
| Zf th1 F | GACGGAAGATGATCGGAGACA |
| Zf th1 R- | CCGCCATGTTCCGATTTCT |
| Zf th2 F- | CTCCAGAAGAGAATGCCACATG |
| Zf th2 R- | ACGTTCACTCTCCAGCTGAGTG |
| Zf tph2 F- | GCAAATACTGGGCTCGGAGA |
| Zf tph2 R- | GAGCATGGAGGATGCAAGGT |
| Zf tph1b F- | GCCTGTTGCTGGCTATCTGT |
| Zf tph1bR- | CTCATGACAGGTGTCCGGTTC |
| Zf tph1a F- | TCTACACACCTGAGCCAGAC |
| Zf tph1a R- | CCCTTCCTGCTTACAGAGCC |
| Zf bdnf F- | CTCGAAGGACGTTGACCTGT |
| Zf bdnf R- | CGGCATCCAGGTAGTTTTTG |
| Zf p75NTR F- | TTAAACGGTGGAACAGTTGTAA |
| Zf p75NTR R- | CTTGACCACTGTAGTGGAG |
| Zf tp53F- | ATGAGGAGATCTTTACCCTGCAG |
| Zf tp53 R- | TGAGGCAGGCACCACATC |
| Zf Δ113p53 F- | ATATCCTGGCGAACATTTGGAGGG |
| Zf Δ113p53 R- | CCTCCTGGTCTTGTAATGTCAC |
| Ntrk2b exon 17 F- | ACTATAGGGTGGGTGGTCACA |
| Ntrk2b exon 17 R- | CTTTGGGGCAAGTACGAGGT |
| Ntrk2b exon 8-13 F- | AGAGGACAGAGGGAACGTGA |
| Ntrk2b exon 8-13 R- | AGACGAGGAGCCTTTCATTCC |

Supplementary table 2- List of antibodies

| Serotonin antibody | S5545, Sigma, St. Louis, MO, USA |
| --- | --- |
| Tyrosine hydroxylase antibody | Diasorin, Stillwater, MN, USA |
| Polyclonal Trk SC-11 | C-14, Santacruz, USA |
| Polyclonal Pan-Trk | A7H6R, Cell signaling, Netherlands |
| Monoclonal TrkB | Clone 47, BD biosciences, USA |
| Actin | Clone AC-74, Sigma-aldrich, USA |
| Monoclonal TrkB | AF1494, R and D systems, USA |
| GAPDH | FL-335, sc-25778, Santacruz, USA |
| Goat anti rabbit -HRP | Cat.no. 1706515, BioRad, USA |
| Goat anti mouse -HRP | Cat.no. 1706516, BioRad, USA |

Supplementary figure 1: Expression analysis of *ntrk2b* and *ntrk2a* at different stages of development.

A: Comparative expression pattern of *ntrk2a* and *ntrk2b* during different stages of development 1dpf, 3dpf, 5dpf and 6dpf.


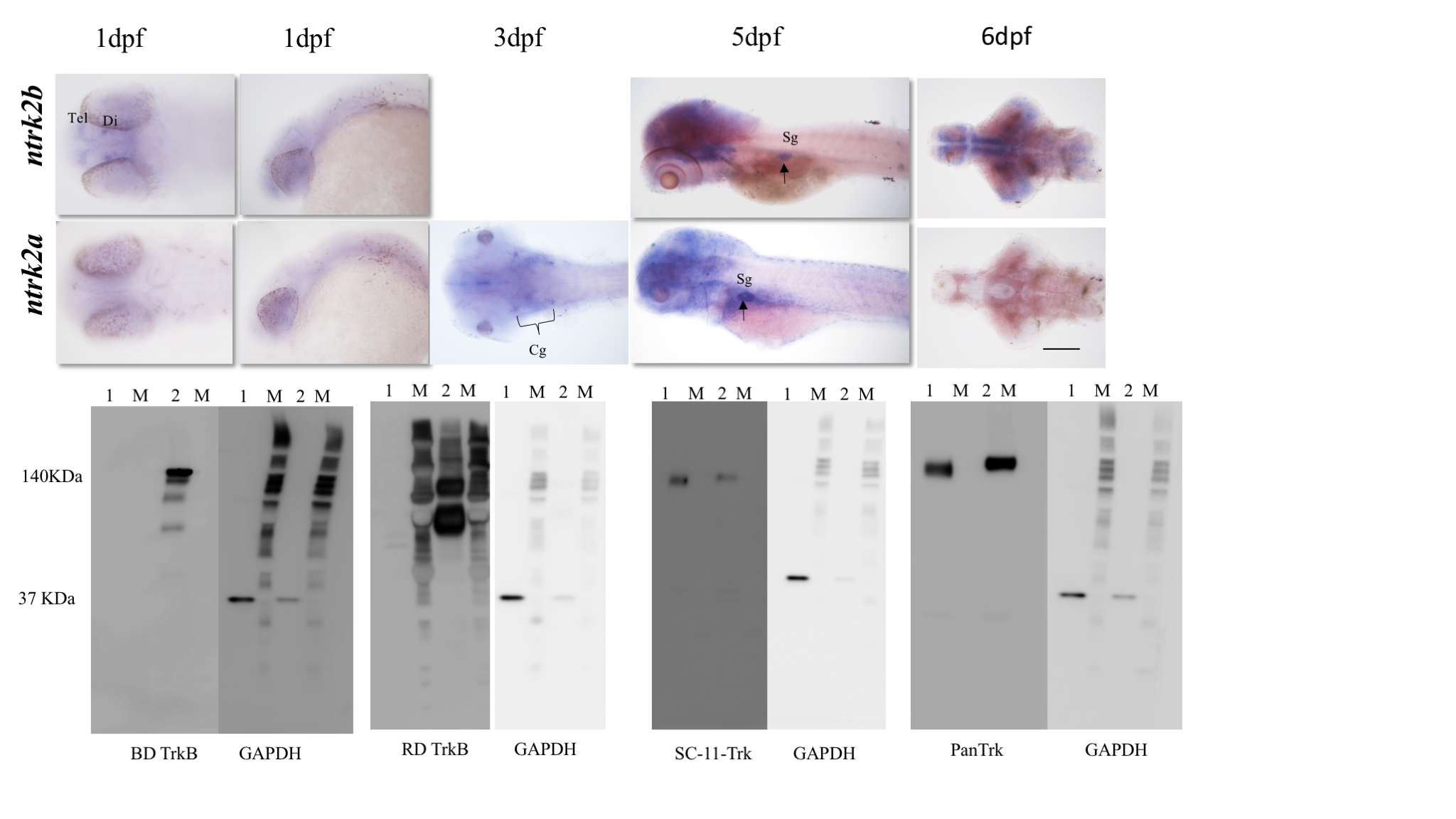


Supplementary figure 2: A- Comparative analysis of different Trk antibodies. The protein levels normalized to GAPDH.

1- adult zebrafish brain lysate, M- Pre-stained marker, and 2- mouse brain hippocampus lysate,


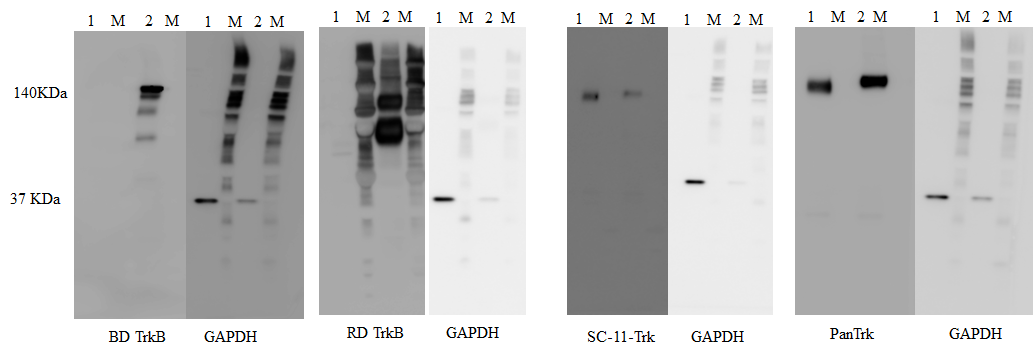


B- Full western blots for A- Whole larval injected morphants, B-Dissected brains from wildtype and Mutants


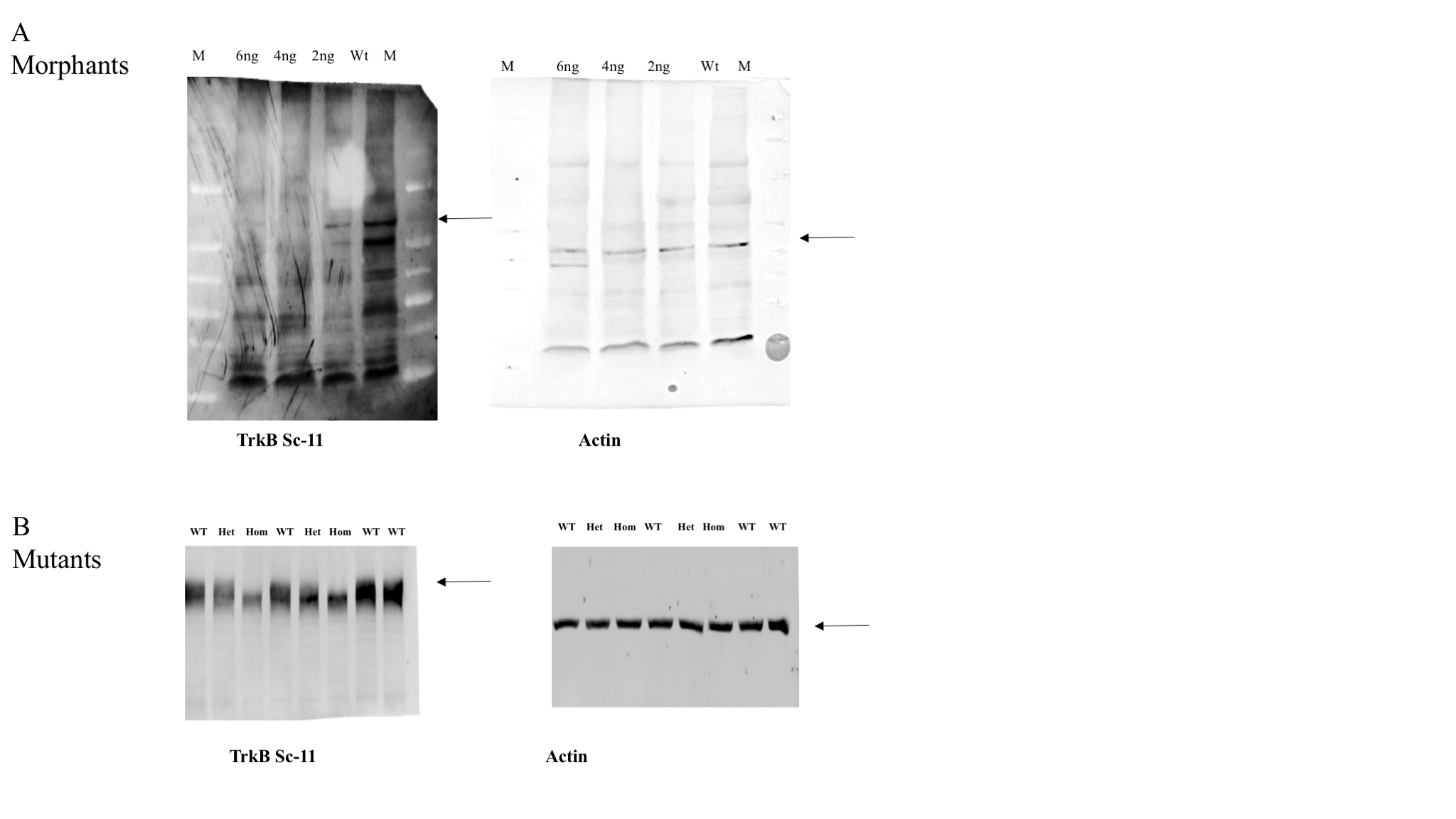


Supplementary figure 3- A: PCR with primers for exon 17, exon 8-13 and ß-actin levels. B: PCR product sequence verified chromatograms to identify wild-type, heterozygous and mutants.

C: PCR products demonstrating specificity of Ntrk2a MO to generate splice variants. D: Gross phenotype of MO injected fish.


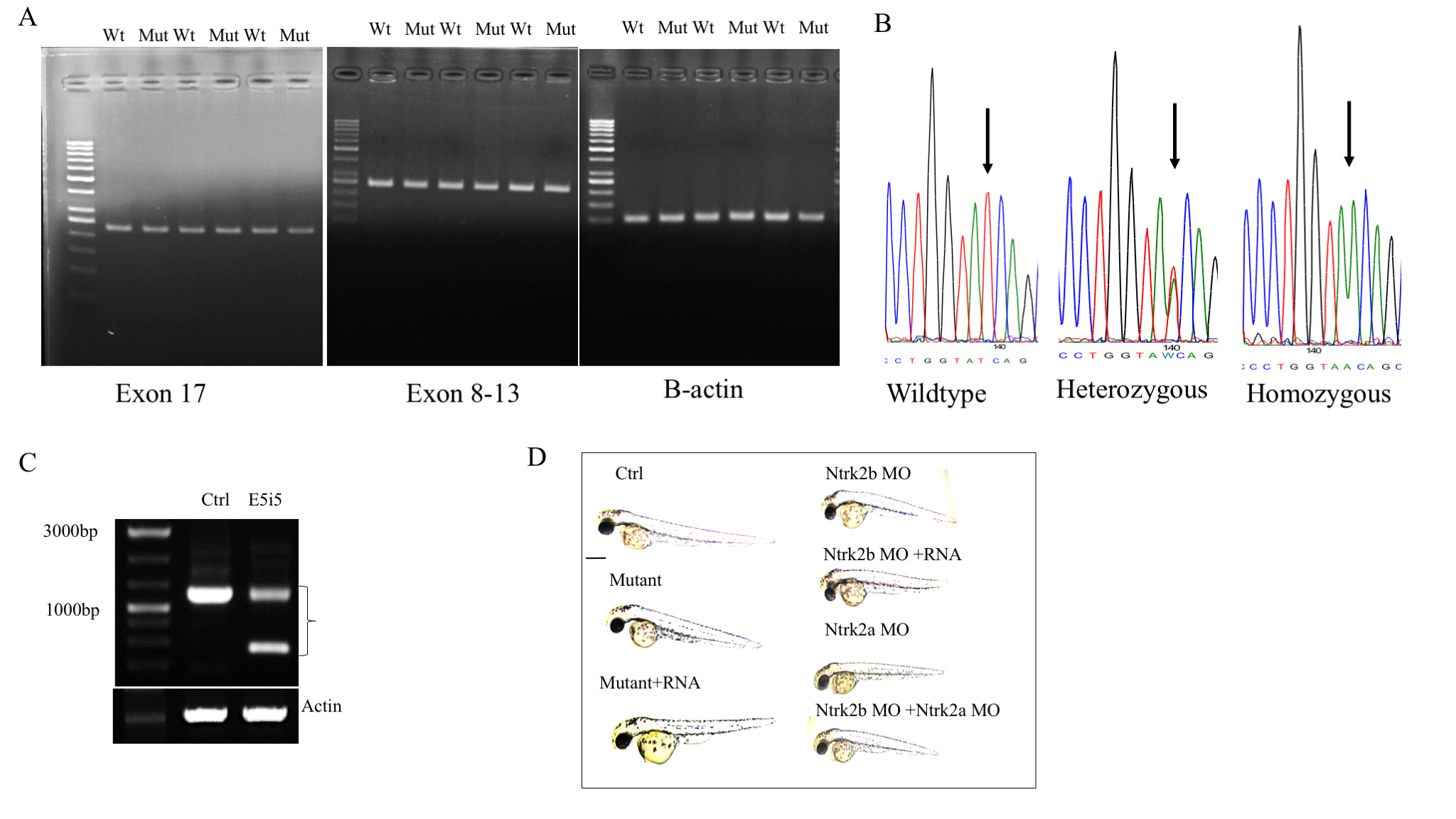


Supplementary figure 4- Wholemount 5dpf brain immunoreactivity for A: tyrosine hydroxylase , B: Serotonin, and C: Histamine.


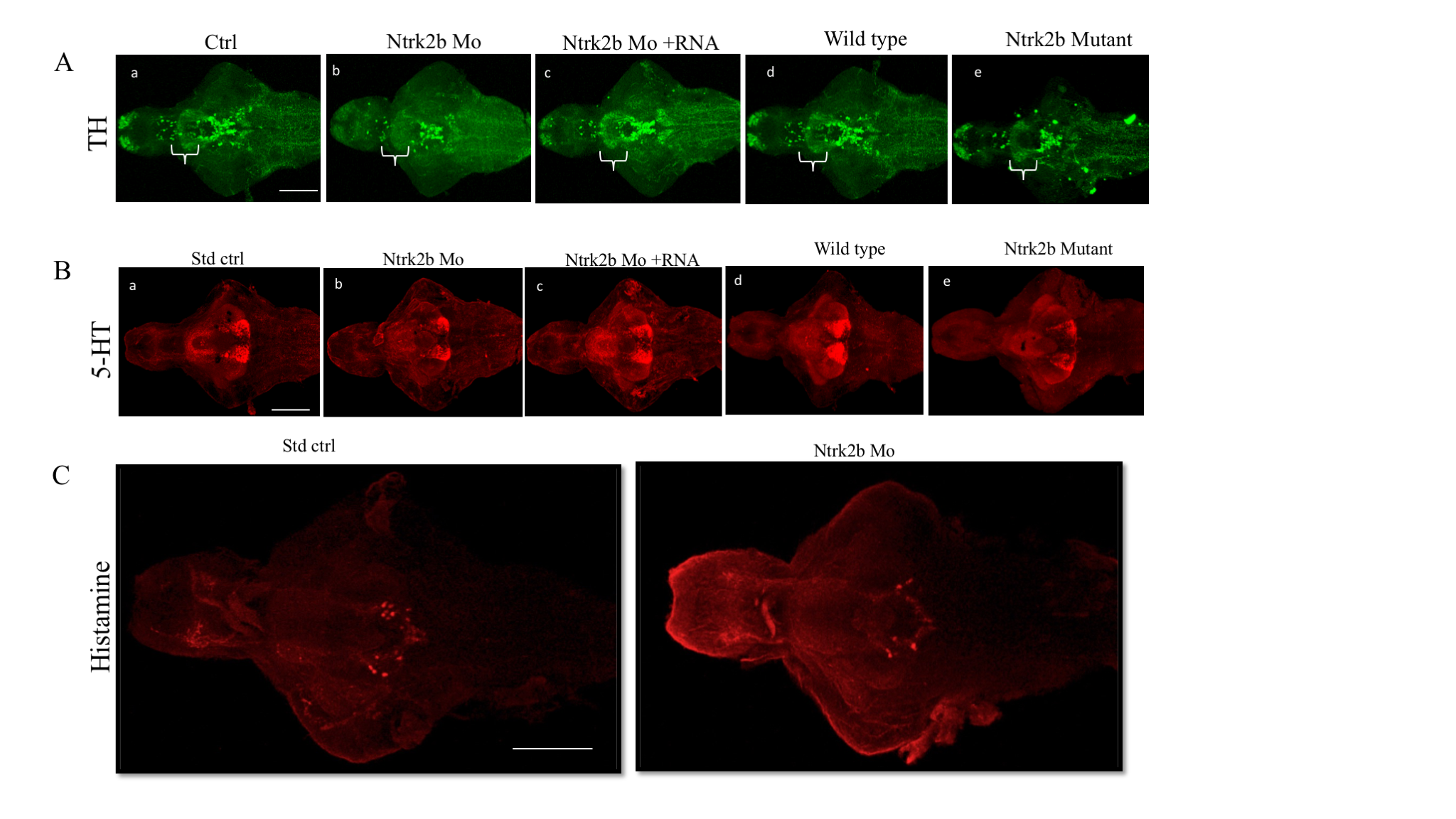


Supplementary figure 5

Full sequence of bdnf clone.

>bdnf

TATGCTCCCGGCCGCCATGGCGGCTCGCGGGAATTCGATTCCCTCGCTCACGGACACTTTCGAGCAGGTCATTGAGGAGTTGCTTGAGGTGGAAGGGGAAGCGACGCAGCAACTGGGGCCTGGGGCCGACCAGGGCCAAGGAGGGGGCGGCCCTATAGATGCGGCAGACTCGAAGGACGTTGACCTGTATGCCTCGCGAGTGATGATCAGCAACCAAGTGCCTTTGGAGCCGCCGTTACTCTTTCTCTTGGAGGAATACAAAAACTACCTGGATGCCGCCAACATGTCGATGCGTGTGCGGCGACACTCGGACCCCGCACGGCGAGGGGAGCTCAGCGTTTGTGACAGTATTAGCCAGTGGGTGACAGCTGTGGACAAAAAGACGGCAATAGACATGTCGGGCCAGACGGTCACCGTTCTGGAGAAGGTCCCCGTGACTAATGGTCAGCTGAAGCAATACTTTTACGAGACCAAATACAACCCCTTGGGGTACACAAAGGAGGGCTGCCGAGGAATAGACAAGCGGCACTATAACTCG
